# MiRNAs in Interstitial Skin Fluid Sampled with Swellable Hydrogel Microneedles Are Locally Deregulated Near Malignant Skin Lesions in Early Stages of Cutaneous Squamous Cell Carcinoma

**DOI:** 10.1021/acsbiomaterials.5c01505

**Published:** 2026-01-22

**Authors:** Ahmad Kenaan, Oliver Teenan, Connor Daniels, Christina Malaktou, Mo Akhavani, Nikolaos Sideris, Leandro Castellano, Jessica Strid, Claire A. Higgins, Sylvain Ladame

**Affiliations:** † Department of Bioengineering, 4615 Imperial College London, White City Campus, London W12 0BZ, U.K.; ‡ Department of Immunology and Inflammation, Imperial College London, Hammersmith Campus, London W12 0NN, U.K.; § The Plastic Surgery Group, 100 Harley Street, Marylebone, London W1G 7JA, U.K.; ∥ Department of Biochemistry, 1948 University of Sussex, Brighton CN1 9QJ, U.K.

**Keywords:** diagnostic biomarkers, microneedles, swellable hydrogels, microRNAs, interstitial skin fluid, skin cancer

## Abstract

Interrogating molecular biomarkers in bodily fluids has emerged as a clinically useful strategy for the early diagnosis of many cancer types. Interstitial skin fluid is currently being explored as a possible alternative to blood, containing the same types of biomarkers but lacking cells and debris that hold little or no clinical value. The discovery and validation of molecular biomarkers with diagnostic or prognostic value and the development of clinical tests based on their detection require minimally invasive technologies capable of sampling this fluid in a pain-free manner. Biomarkers must also be easily recoverable for follow-on analysis. Herein, we combine standard genomic approaches with innovative bioengineering technologies to demonstrate that short noncoding miRNAs are significantly deregulated in extracellular skin fluid surrounding malignant skin lesions, providing a yet largely unexplored window of opportunity for early diagnosis of skin cancers. Hydrogel-based microneedle patches offering clinically useful sampling capacity were developed that enable the rapid capture and recovery of endogenous miRNAs from human skin through deformation of the epidermal–dermal junction. Using mouse models of cutaneous squamous cell carcinoma, a significantly greater level of deregulation of selected miRNAs was observed in perilesional skin fluid compared to that in blood levels.

## Graphical abstract

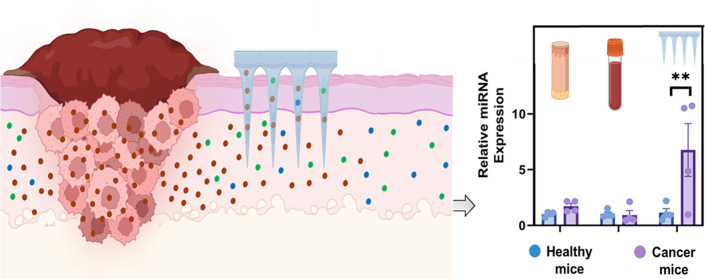

## 1 Introduction

Skin cancer diagnosis is most commonly derived from visual or digital inspection of a skin lesion by a trained professional, ideally including dermoscopy. Identification of suspicious skin lesions in primary care is typically followed by an urgent referral to secondary care, leading to a skin biopsy and histopathological examination, in suspicious cases.^1^ While primary care clinicians are generally accurate at recognizing suspicious skin lesions (with melanoma having one of the lowest median primary care intervals), only 6–15% of patients referred to secondary care end up being diagnosed with skin cancer.^2,3^ A technology that could help general practitioners (GPs) more effectively screen out patients who do not require dermatological assessment and biopsy would allow significant savings and reduce the level of unnecessary morbidities.^4^

Various transcriptional profiles have been identified that could accurately distinguish between benign nevi and melanoma with clinical applications including diagnosis and prediction of recurrence. Biomarker panel sizes range from as little as 2 for the pigmented lesion assay (PLA, DermTech, USA) and up to 31 for Decision-Dx-MelanomaTM (Castle Biosciences, USA). Biological specimens include cell-debris collected from sticky patches (for DermTech) and tissue biopsies (for Castle).^5^ Recently, researchers have identified a number of noncoding miRNAs deregulated in bodily fluids of patients with various forms of skin cancer, including melanoma.^6^ Individual miRNAs and miRNA signatures in blood, in patients with melanoma, can diagnose disease with a sensitivity comparable to that of a skin biopsy (e.g., up-regulated miR-149-3p and miR-150-5p and downregulated miR-193a-3p, AUROC = 0.97).^7^ While blood and urine are commonly the two most interrogated bodily fluids,^8^ their analysis is frequently insufficient to disclose localized tissue deregulation.^9,10^ Within the skin dermis, interstitial skin fluid (ISF), also known as tissue fluid, resides between cells and has recently emerged as a possible alternative for liquid biopsy sampling.^11^ Its molecular composition resembles that of the initial lymph. In addition to electrolytes and plasma proteins, it also contains substances which act on the tissue in addition to those which are carried to other organs’ interstitium via blood circulation.^12^ ISF is a much less complex matrix when compared to blood plasma or serum, which greatly facilitates the detection of molecular metabolites.^13,14^ Until recently, analyses of the molecular components of ISF have mainly focused on the proteome.^15^ However, a recent study used Next Generation Sequencing to analyze matching samples of dermal ISF, serum, and plasma with many types of potentially informative RNA species detected in all three biofluids.^16^ The RNA species identified included mRNA’s (or mRNAs), long noncoding RNAs (or lncRNAs), and short noncoding microRNAs (or miRNAs).

Minimally invasive technologies for ISF sampling have emerged that are based on compact patches of microneedles (MNs).^17^ They are typically made of an array of microscale solid, porous, or hollow needles, from materials such as glass, metal, silicon, or other polymers.^18^ They represent very useful alternatives to ISF sampling technologies currently used in clinical practice.^19,20^ These invasive technologies rely on skin removal (tissue biopsy), skin puncture (microdialysis and dermal open-flow microperfusion), or applied suction (suction blister) and cannot be implemented in primary care facilities, such as GP surgeries. They are all painful to the patient, potentially leading to increased anxiety and decreased compliance with future medical procedures and are prone to skin inflammation or skin infection.^21,22^ MN patches represent a promising alternative for biomarker extraction and detection from ISF. However, a large proportion of technologies developed to date typically suffer from a too low sampling capacity (<2 μL) and too slow sampling rate to enable a comprehensive analysis of skin ISF. Higher sampling capacity at a faster rate would enable us to investigate the near real-time dynamic of ISF composition in response to various stimuli.^17,23^ Hydrogel microneedles (HMNs) have so far mostly been developed and applied for drug delivery applications where swelling of the hydrogel matrix upon insertion into skin and absorption of skin fluids increases the material’s mesh size, leading to controlled release of its payload into systemic circulation.^24,25^ Only recently have HMNs also been exploited for their “absorbent” properties, offering a rapid and effective way to sample ISF painlessly, minimally invasively, and with high spatiotemporal resolution. This could pave the way for the next generation of point-of-care diagnostic devices.^26^ Specific requirements for these applications include strong mechanical properties (to safely induce skin penetration or deformation), biocompatibility (to minimize adverse responses, inflammation, or tissue damage), and high swelling ratio (to collect sufficient amounts of fluid for subsequent biomarker analysis). Also very important and often overlooked is the necessity for captured ISF biomarkers to be released from HMNs efficiently and under mild conditions that do not affect their integrity. This is particularly essential, and challenging, for long RNA biomarkers (coding and noncoding) that have limited stability under nonphysiological conditions of salt and/or pH, and to a lesser extent to small noncoding RNAs (including miRNAs) which, in comparison, have a greater ability to withstand changes in pH and temperature.^27^ Herein, we developed and characterized a small library of HMNs tailored for the sampling and efficient recovery of miRNAs from ISF. We demonstrate that noncytotoxic HMNs can rapidly absorb ISF miRNAs from human skin, without penetration through the epidermal/dermal junction required. Further still, ISF biomarkers can be efficiently recovered post sampling under mild conditions suitable for follow-on miRNA extraction (using commercial extraction kits) and RT-qPCR analysis.

Using mouse models of cutaneous squamous cell carcinoma (cSCC) and hypothesis-free small RNA-Seq, we first show that miRNAs are significantly deregulated in the ISF of cSSC mice compared to healthy mice. We then demonstrate that a selected panel of miRNAs sampled with our HMNs near emerging skin lesions are deregulated in mice with induced cSCC compared with healthy mice and that significantly greater levels of deregulation are observed in HMN-extracted fluid compared to blood or even tissue from cSCC mice. This work validates skin fluid as an easily accessible source of clinically useful molecular biomarkers and swellable HMNs as next generation medical devices for improving diagnosis of skin diseases, including skin cancers, in primary care.

## 2 Materials and Methods

### 2.1 Materials

The polydimethylsiloxane (PDMS) female mold was produced as previously reported by the Irvine lab.^28^ Briefly, it presents an array of 9 × 9 microneedles of pyramidal shape with the following parameters: height = 550 μm; base dimension = 0.25 mm × 0.25 mm base; spacing between needles = 0.34 mm; spacing between rows = 0.4 mm. Poly­(vinyl alcohol) (PVA, *M*w = 27 kDa), sodium carboxymethyl cellulose (CMC, *M*w = 90 kDa), 5(6)-carboxyfluorescein, and 1× penicillin/Streptavidin were purchased from Sigma-Aldrich Chemistry Co., Ltd. (UK). Chitosan (CS, 200–600 mPa·s, 0.5% in 0.5% Acetic Acid at 200C) was purchased from Tokyo Chemical Industry Co., Ltd. (Belgium). Polyvinylpyrrolidone (PVP, *M*w = 40 kDa), acetic acid (0.1 N), glutaraldehyde (50% aq soln.), genepin (GP), citric acid (CA, 99.6%), collagenase I, dispase II, and TaqMan-probe were purchased from Thermofisher scientific Co., Ltd. (UK). Serum/Plasma Advanced kit and miRNeasy mini kit were purchased from QIAGEN, LLC (Germany). miRNA cDNA synthesis kit, TaqMan Universal Mater Mix II (no UNG), and Quantstudio 3 were purchased from Applied Biosystems, LLC (USA). 10% FBS and DMEM F-12 were purchased from Gibco, Ltd. (UK). Alamar Blue Assay was purchased from Invitrogen Co, Ltd., (USA).

### 2.2 HMN Patch Preparation

#### 2.2.1 General Fabrication Procedures

The HMN patches were prepared by micro molding. Briefly, PVA blends were cast into a female PDMS mold and cross-linked by either addition of a chemical cross-linking agent or undergoing repeated freezing and thawing cycles. To remove any air bubbles, molds filled with PVA/PVP/CS and PVA/CS blends were centrifugated while molds filled with PVA/PVP and PVA/CMC blends were degassed under vacuum. All HMN patches were finally cured at 60 °C for 6 h and left to cool down at room temperature before they could be peeled off.

#### 2.2.2 PVA Blends’ Preparation and Cross-Linking

Aqueous PVA solutions were prepared at either 24 wt % (for PVA/CMC and PVA/PVP blends) or 15 wt % for the PVA/CS and PVA/PVP/CS blends. Aqueous stock solutions of PVP and CMC were also prepared at final concentrations of 18 and 10 wt %, respectively. A stock solution of 2 wt % Chitosan was prepared in 0.1 M acetic acid by stirring at room temperature for 6 h. For chemical cross-linking, PVA, PVP, and the cross-linking agent casting solutions were prepared as follows: PVA and PVP were mixed in a 1:1 volume ratio. Then, various cross-linking agents were added, including GA (4 v/v %), GP (0.1 and 1 wt %), STB (2 wt %), and CA (10 wt %). For the PVA/CMC blend, only GA (4 v/v %) was tested. After thorough mixing, the different PVA blends were left under stirring at room temperature overnight before casting into the PDMS molds. 12 wt % PVA solution was prepared and then subjected to cross-linking with 4 v/v % of GA as a control experiment.

For physical cross-linking, PVA/CS (1:1, v/v) and PVA/PVP/CS (1:1:1, v/v/v) blends were prepared, added into the PDMS molds, and subjected to repeated cycles of freezing and thawing. Each cycle consisted of overnight freezing at −20 °C and slow thawing at room temperature for 6 h, and this cycle was repeated twice (for a total of three cycles) to ensure complete and homogeneous physical cross-linking of the hydrogel.

### 2.3 HMN Patch Characterization

#### 2.3.1 Swelling Properties

HMN patches were submerged in distilled water and left to soak for 15 min. The patches were then carefully removed from the fluid and any excess surface liquid gently removed. The weights of both the dry and swollen HMNs were recorded and the swelling ratio calculated using eq 1

1 $$swelling\;ratio=\left(m_{f}-m_{i}\right)/m_{i}\times 100$$

where *m*_f_ is the mass of the swollen patch and *m*_i_ is the mass of the dried patch.

#### 2.3.2 Stiffness Measurements

To assess the mechanical properties of the prepared HMN patches, measurements were conducted using an ElectroForce 5500 test instrument. HMN patches were placed between two plates. A 200 N load cell was used to apply a compressive force at a constant rate (0.02 mm/s) until the microneedles start to deform or break. The force (load) versus deformation (displacement) curves were recorded and converted to stress–strain. Calculating the slope of the linear portion of the stress–strain curve provided the Young’s modulus (*E*) given by eq 2

2 E=σ/ε

where σ is the stress percentage, or the amount of force applied per unit area (σ = (*F*/*A*)), *A* is the area of the MN patch, and *F* is the loading (Force); ε is the strain, it is the extension per unit length (ε = 100 × (dl/*l*)), where dl is the displacement (deformation), and *l* is the length of the MN patch. To ensure accurate and reliable results, each experiment was repeated six times for every individual patch material.

### 2.4 Skin Penetration Testing and H&E Staining

Human skin was obtained from patients undergoing abdominoplasty surgery after written informed consent was taken using consent forms approved by the Imperial College Research Ethic Committee. The skin was then stored in an Imperial College Healthcare Tissue Bank (ICHTB) subcollection and used in an ICHTB approved project. Skin was collected, fat was removed, and the skin was cleaned with iodine and then dried. For dermal staining, microneedles were pressed into the skin surface, then removed and trypan blue was added to the site. This was allowed to stain tissue for 2 min before being removed by washing the skin surface with 1× PBS. The skin was then dried before macroscopic images were taken on a Leica Stereo microscope. For H&E staining, small squares of 1.5 cm × 1.5 cm were cut from the skin before microneedles were placed onto the skin epidermis and pressure applied to the top. Microneedles were held with pressure from forceps and placed into an OCT mold and covered in OCT ensuring constant application of pressure. Molds were then placed in dry ice and methanol for rapid freezing with the microneedle in situ. 20 μm sections of skin were cut from OCT blocks and mounted onto slides for H&E staining. H&E was performed as follows: fixed in 10% formalin for 10 min, washed in distilled water, 4 min Haematoxylin, washed in PBS, 0.3% acid alcohol for 5 s, Scott’s Tap water for 15 s, and then washed again in PBS; Eosin for 2 min, then dehydrated in 70% ethanol, 90% ethanol, and 100% ethanol, and then washed twice in Histo-Clear before mounting with DPX. All images were taken on a Leica DMi1 microscope.

### 2.5 Cell Viability

Primary human fibroblasts were isolated from human skin samples after enzymatic digestion of the dermis and fibroblasts allowed to proliferate in a 6-well plate. Cells were cultured in 10% FBS, DMEM F-12, and 1× Penicillin/Streptavidin. Cells were seeded at full confluence in a 24-well plate. After 24 h, they were placed in a 0.4% serum media before microneedles were placed needle tips down in the plate. Microneedle patches were left for 10 min at 37 °C and then removed with forceps, and the media was aspirated off and replaced with phenol red free media. Cell viability was assessed with the Alamar Blue Assay with untouched cells measured as 100% viable cells and 0% viable cells measured after triton-X treatment of cells for 10 min. Alamar blue was added to media on cells and incubated at 37 °C for 4 h; absorbance was then measured at 590 nm (BMG Labtech, Germany).

### 2.6 Chemical Cutaneous Carcinogenesis Mouse Models

BALB/c wild-type (WT) mice were purchased from Charles River and were maintained in individually ventilated cages under specific pathogen-free conditions. Mice were age-matched and used at >7 weeks of age. The study complied with Imperial College AWERB (Animal Welfare and Ethical Review) guidelines and UK Home Office regulations. Cancer growth strictly adhered to the guidelines for Welfare and Use of Animals in Cancer Research (P. Workman et al., British Journal of Cancer (2010) 102, 1555–1577). The chemicals 12-0-tetradecanoylphorbol-13-acetate (TPA; Sigma-Aldrich) and dimethylbenz­[*a*]­anthracene (DMBA, Sigma-Aldrich) were dissolved in 100% ethanol or acetone, respectively. For carcinogenesis induction, age-matched female mice at 7 weeks were used and the back skin was shaved using hair clippers 1 week before carcinogen initiation. 600 nM DMBA was carefully applied by pipetting in 100 μL volume to the entire shaved back skin area and mice were then rested for 1 week. This was followed by twice-weekly application of 20 nM TPA in a volume of 100 μL on the entire back for 8 weeks. Hair regrowth was gently removed using hair clippers. At 8 weeks, the experiment was terminated, and skin tissue and blood collected. Blood was spun down at 6000*g* for 10 min and serum was collected. Serum was stored at −80 °C until used.

### 2.7 miRNA Sampling, Recovery, and Analysis

#### 2.7.1 Skin Fluid Collagenase Extraction from Human Skin

Punch biopsies were taken from abdominal skin (3 mm) and then placed in Collagenase I (1 mg/mL) for 24 h at 4 °C. ISF was then centrifuged through a 10 μm filter to remove any cellular content, and the top portion was taken to ensure cells and cellular debris were not included in the sample.

#### 2.7.2 Skin Fluid Sampling and Recovery Using HMNs

HMN patches were submerged in ISF solution and allowed to soak for 15 min. Removed patches were then rinsed with DI water, and any excess surface liquid was carefully removed. The swollen patches were then placed (needle tips down) in a 24-well plate containing 1 mL of DI water and heated at 60 °C for 15 min. A 200 μL solution was collected and used in PCR following RNA extraction to detect the presence of specific miRNAs.

#### 2.7.3 ISF Sampling from Human Skin Using HMNs

HMN patches were placed on fresh skin biopsies from abdominoplasty surgery, and firm thumb pressure was applied. After 10 min, the patches were carefully removed and then placed (needle tips down) in a 24-well plate containing 1 mL of DI water and heated at 60 °C for 15 min. A 200 μL solution was collected and subjected to PCR testing following RNA extraction to detect the presence of specific miRNAs.

#### 2.7.4 miRNA Extraction

Total miRNAs were extracted from skin fluid using the miRNeasy Serum/Plasma Advanced kit following the manufacturer’s protocol. Prior to extraction, 33 fmol of *C. elegans* miR-39 (Norgen, Ontario Canada) was added to each sample for normalization. To prevent the hydrogel from obstructing the filters, the samples were consistently centrifuged at 17,000*g* during extraction. The extracted total RNA was then eluted in 20 μL of RNase-free water. For skin fluid RNA extraction, 200 μL of skin fluid was diluted to 1 mL in water, and the extraction process was carried out as described above. In the case of dermis tissue RNA extraction, a 1 mm biopsy from the dermis was manually homogenized by using a syringe in Qiazol. Subsequently, the miRNeasy mini kit was used to extract RNA from the homogenized sample, following the manufacturer’s instructions. For extraction from mouse skin tissue, 3 mm skin biopsies were taken and homogenized in Qiazol and total RNA extracted using the miRNeasy mini kit.

#### 2.7.5 miRNA Analysis by RT-qPCR

1–2.5 μL RNA (1–2.5 μL) was used and loaded neat into each reaction in the TaqMan MicroRNA Reverse Transcription kit. Quantitative PCR was performed using the TaqMan Universal Mater Mix II, no UNG instrument, and TaqMan-probe. Assay IDs: miR-21 000397, miR-205 000509, miR-125b 000449, miR-26b 000407, miR-146 001097, miR-30d 000420, miR-39 000200, U6 001973. Quantitative PCR was performed on the Quantstudio 3, miR-39 was used to normalize liquid biopsy samples, and U6 was used for tissue. All statistical analysis was performed on ΔCT, in GraphPad Prism, 10.

### 2.8 Bioinformatic Analysis of ISF

Total RNA was extracted as previously described and submitted to Lexogen for small RNA-sequencing. Raw reads were examined by FASTQC, before being trimmed and filtered by quality score using the miRDeep2 package,^29^ aligning to GRCm39 to generate a counts matrix. Differential expression analysis was performed with DESeq2 (Version 1.46.0) in RStudio, log fold change shrinkage using apeglm was performed to account for expected high variability, and minimum mean counts across samples >50, significance was set to *p* < 0.05, fold change >1.5.

## 3 Results and Discussion

### 3.1 miRNA Biomarker Identification

Initial studies aimed to provide a proof of concept of changes in miRNA expression in the skin interstitium across different skin sites using a mouse model of cSCC. To induce carcinogenesis, mice were exposed topically to the carcinogen dimethylbenz­[*a*]­anthracene (DMBA) on shaved back skin. Outgrowth of mutated epithelial cells was thereafter promoted by twice weekly topical application of the inflammatory agent 12-o-tetradecanoylphorbol-13-acetate (TPA). Mice were subject to 8 weeks of TPA application before skin biopsies were performed adjacent to visible tumors (Tumor skin), 2 cm adjacent to the tumor (Perilesional skin), or from nontreated skin sites (*N* = 3). ISF was released from biopsies through enzymatic treatment, collagenase I at 4 °C overnight, before being centrifuged through a 10 μm filter, and the remaining fluid was collected from the flow through. miR-21 and miR-205, 2 well described markers of cSCC, were then quantified by qPCR (Figure 1A). Significantly higher levels of miR-21 and miR-205 were measured in perilesional skin compared to nontreated skin, and miR-21 was also measured as higher in tumor skin. This provided early validation that ISF miRNome does indeed reflect the health of skin tissue and that spatial differences in ISF arise within an organism. Next, a preclinical study was established to profile the ISF miRNome. Mice were subjected to 8, 12, or 17 weeks of TPA application along with naïve control mice. 3 mm skin biopsy samples were taken from perilesional sites, ensuring samples did not visually contain tumors, and ISF was released with collagenase I treatment. RNA was extracted, quantified, and concentrations normalized for small RNA-sequencing (Figure 1B).

**Figure 1:**
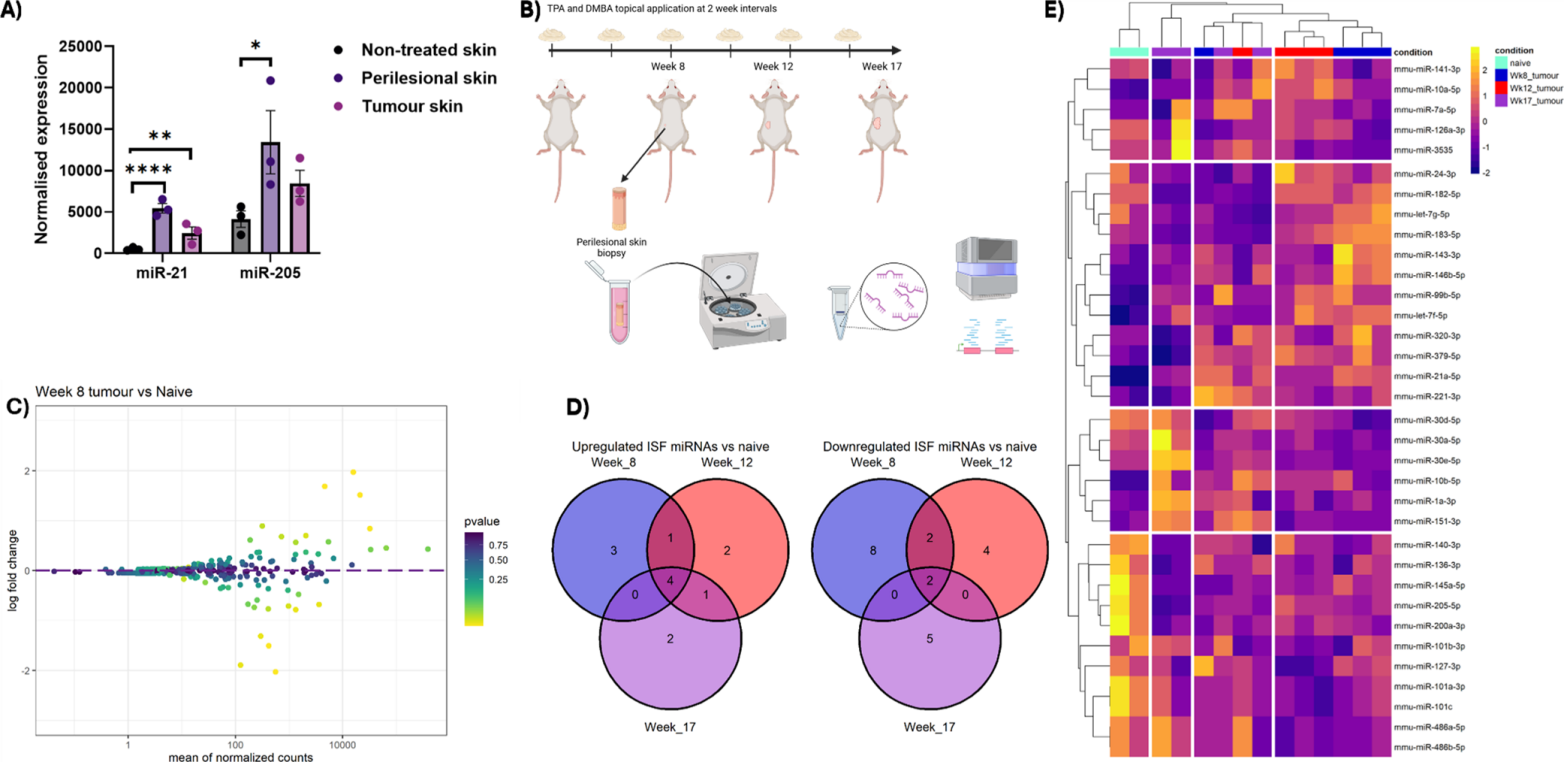
(A) qPCR analysis of ISF isolated from different sites on a tumor induced mouse. Skin was sampled from a nontumor, perilesional (2 cm away), and directly adjacent to the tumor. ISF was extracted by collagenase extraction, and miRNA was extracted and quantified by qPCR. miR-21 and miR-205 were measured and normalized expression plotted relative to miR-39. (B) Schematic depicts the workflow for SCC modeling, followed by the enzymatic ISF extraction. Mice were culled at week 8, week 12, and week 17 of TPA and DMBA application, skin biopsies were taken avoiding any visible tumor, along with the serum for further validation. Skin biopsies were placed in collagenase I (1 mg/mL) for 24 h before the tissue and eluate was centrifuged through a 10 μm filter and RNA was extracted and quantified before being submitted for small RNA-sequencing. (C) Small RNA seq was performed on collagenase-extracted ISF, DESEq2 analysis highlighted top differentially expressed miRNAs in ISF after log fold shrinkage, comparison shown in Week 8 tumor mice vs naïve, Log fold change against normalized counts, p-value shown by color. (D) The Venn diagram shows the number of ISF miRNAs significantly deregulated compared with naïve controls for each tumor time point, measured by DESeq2. (E) *z*-scores were calculated for significantly deregulated miRNAs from each time point, compared with naive controls; samples were clustered by K-means clustering and cut into 4 groups.

Small RNA-sequencing generated Fastq files for each sample, and these were filtered and aligned using miRDeep2 to generate a counts matrix of 332 miRNAs with sufficient counts for analysis. The DESEq2 package was used in R to identify differentially expressed miRNAs between each time point and the naïve untreated samples. Twenty significantly deregulated miRNAs were present after 8 weeks of TPA application compared to naïve controls, including increased miR-21a, miR-143, and miR-146b concentration (Figure 1C). After 12 weeks of DMBA application, 16 miRNAs were significantly deregulated in ISF, with increased abundance of miR-21a, let-7f, and miR-10a. At 17 weeks of application, 14 miRNAs were significantly deregulated, miR-183 and miR-182 concentrations were measured and found to be decreased, while miR-21a and let-7f were significantly increased (Figure S1). Changes in ISF composition from week 12 to week 17 were also measurable, with 8 miRNA altering the expression between the two time points, miR-183 and miR-182, again significantly underrepresented in the later time point along with miR-203. Finally, all tumor time points were grouped together and compared with naïve mice. This highlighted only 6 consistently deregulated miRNAs, including miR-21a, let-7f, and miR-146b overexpression, with loss of miR-101a. A Venn diagram highlights the number of differentially abundant miRNAs measured within the ISF at each stage of the disease vs naïve controls, highlighting the consistent upregulation of 4 miRNAs (miR-21a-5p, miR-146b-5p, let-7f-5p, miR-99b-5p) and the consistent downregulation of miRNA (miR-101) (Figure 1D). Significantly deregulated miRNAs compared with naïve controls were subset from the data set, *z*-scores were calculated, and samples were allowed to cluster in a heatmap. This highlighted the separation of naïve control mice across the 34 significantly deregulated miRNAs especially with the 8 week TPA mice (Figure 1E). Due to the strong stratification and improved translatability for early time point biomarkers, 8 week TPA application was selected for validating new, microneedle-based noninvasive technologies for ISF sampling and analysis. While this preclinical model is well characterized, further translational studies would be required to bridge between the preclinical model and human disease. We envisage some of the miRNA biomarkers deregulated in the disease would mirror those important in human disease; however, validation of these targets and the biologically relevant concentrations would still be required for the technology to be transferred into the clinic.

### 3.2 HMN Preparation and Characterization

First, we created six different microneedles to evaluate their potential to absorb ISF. PVA has been extensively used in biomedical applications, especially in the making of HMNs where swellable, water-soluble, and biocompatible polymers are required.^30^ However, hydrogels solely made of PVA, following physical or chemical cross-linking, do not meet the necessary mechanical and swelling requirements for creating functional HMNs, which can be improved by using combinations of polymers.^31,32^ Herein, four different PVA blends were prepared by addition of PVP, CMC, and/or CS in varying ratios (optimized in our laboratory) and cast in female PDMS molds. Gelation was triggered through chemical or physical cross-linking. Glutaraldehyde (GA) and reportedly less toxic genepin (GP) were used for chemical/covalent cross-linking while physical cross-linking proceeded via three consecutive freeze–thawing cycles (Figure 2A,B). After a final curing step, both swelling and mechanical properties of the HMN patches were systematically assessed (Figure 2C,D). Most polymer blends showed improved swelling and mechanical properties when compared to the GA-cross-linked PVA-only HMNs. The highest swelling ratio (defined as the % mass increase upon absorption of water) was obtained with the HMN formed from the cross-linked PVA/PVP and PVA/CMC blends with values of 460.1 ± 57% and 633.3 ± 27%, respectively (Figure 2C). Substituting GA with GP resulted in a c.a. 2-fold drop in swelling capacity. Although GA is one of the most used bifunctional cross-linking agents to create stable biomaterials in biological sciences, its high reactivity and intrinsic cytotoxicity could prevent the clinical implementation of devices based on materials cross-linked in this way. To eliminate the need for chemical cross-linking agents, we explored the formation of HMNs from similar polymer blends through physical cross-linking. This method takes advantage of the unique behavior of water and the polymer chains under freezing temperatures when ice crystals form and push the polymer chains apart. Upon thawing, the polymer chains come back together, creating highly stable physical cross-links through hydrogen bonding and polymer chain entanglement. HMNs formed from blends of PVA and CS via repeated freeze–thawing cycles showed greater swelling capacity than GA-cross-linked PVA on its own (287.6 ± 57.5% and 181.9 ± 45%, respectively). Most interestingly, the incorporation of PVP within the PVA/CS blends increased the swelling ratio up to 481.5 ± 89.3%, reaching a level comparable to that of the PVA/PVP blend chemically cross-linked with GA.

**Figure 2:**
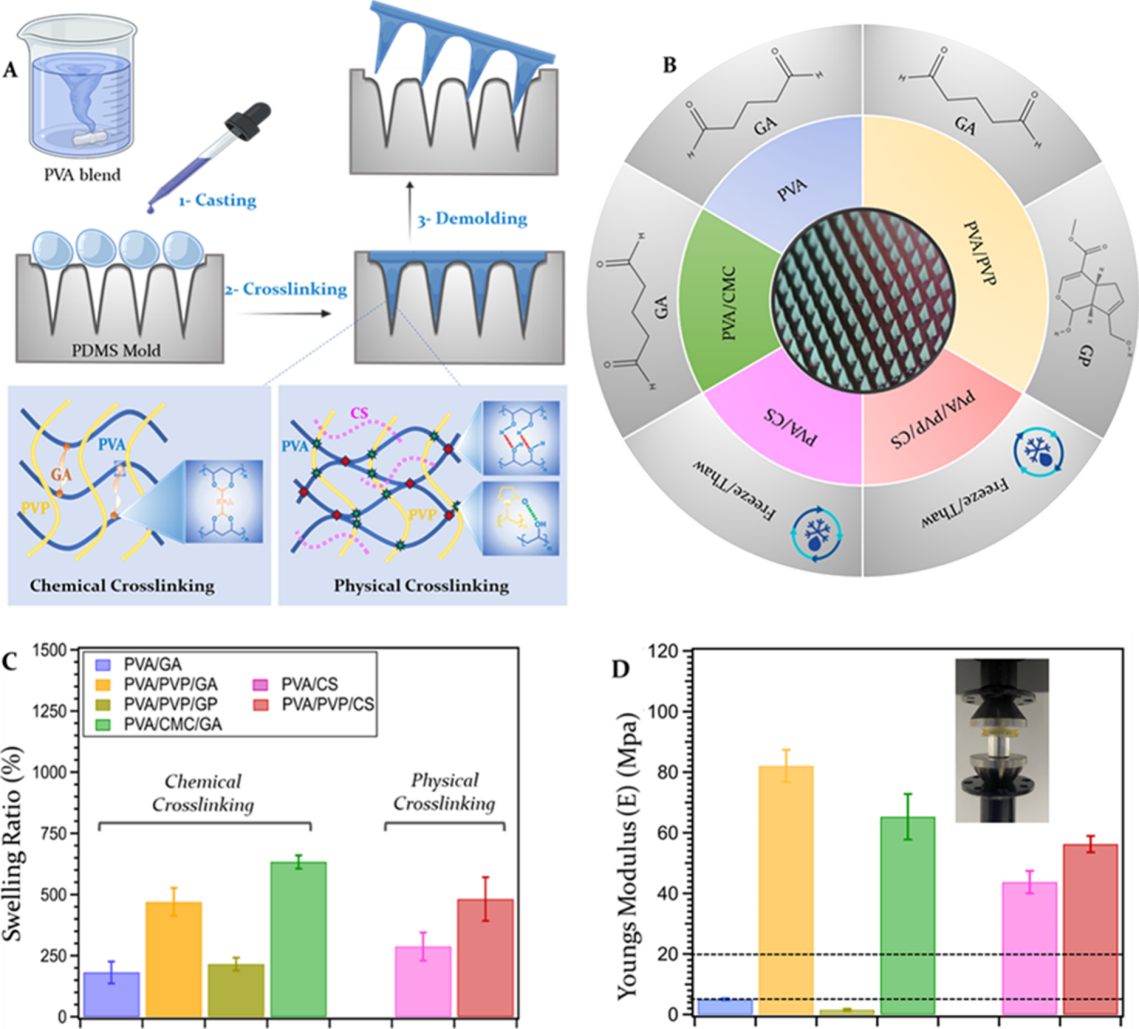
(A) Schematic representation of the HMN preparation process via micro molding followed by chemical or physical cross-linking of PVA blends with PVP, CMC, and/or CS. (B) Schematic representation of the various combinations of polymer blends (inner circle) and cross-linking strategies (outer circle) for HMN fabrication. (C) Swelling ratio evaluation of HMN patches produced via molding technology. (D) Determination of Young’s Modulus values for chosen HMN patches derived from stress–strain curves. The dashed lines indicate the range of Young’s modulus values reported for human skin.^33^ The results are expressed as mean ± standard error of the mean (*n* = 5), with n indicating the number of experimental repeats.

### 3.3 HMN Mechanical Strength

Prior to investigating the impact HMNs have on human skin, their mechanical properties and stiffness were investigated to ensure that they would not bend or fracture during skin insertion. A compressive force (up to 150 N) was applied to the microneedles, and the obtained loading-displacement curves were converted into stress–strain curves. The highest Young’s modulus values (indicating greater stiffness and resistance to deformation) were obtained for GA-cross-linked PVA/PVP and PVA/CMC blends. Interestingly, HMNs made from physically cross-linked blends (PVA/CS or PVA/PVP/CS) also exhibited great stiffness with Young’s modulus values of 48 and 56 MPa, respectively (Figure 2D). The mechanical resilience of the PVA/CS blend is attributed to the formation of intermolecular hydrogen bonds among its various components (PVA–OH==HO-PVA, PVA–OH==HO–CS, and CS–OH==HO–CS). These values are only moderately lower than that obtained with GA-cross-linked PVA/CMC blend (65 MPa). Remarkably, the introduction of PVP into PVA/CS blends further enhances their mechanical strength due to additional hydrogen bond formations involving PVA–OH==HO-PVP and CS–OH==HO-PVP interactions. These interactions contribute to improved cohesion, resulting in a microneedle structure that is both stronger and more unified. Overall, four out of six HMNs showed a Young’s modulus value significantly higher than that reported for human skin (between 4.6 and 20 MPa, highlighted with dotted lines in Figure 2D),^33^ suggesting their ability to overcome skin elasticity and their suitability for application on human skin. These four were therefore selected for further penetration testing on human skin.

### 3.4 Skin Penetration and miRNA Sampling and Recovery

#### 3.4.1 MiRNA Sampling/Recovery In Vitro

Next, the ability to efficiently recover detectable levels of ubiquitous miRNAs from HMNs after sampling was investigated. ISF was first collected from human skin using an optimized collagenase I extraction protocol, where 3 mm punch biopsies were taken from excised abdominal skin, incubated at 4 °C overnight, and centrifuged through a 10 μm filter to remove intact cells. For each HMN patch, biomarkers were collected by being soaked in extracted ISF for up to 15 min at room temperature. Although swelling was not measured directly, no differences were expected with enzymatically extracted ISF. miRNA levels recovered from the HMN after soaking were therefore used as a proxy for swelling and release. The ISF-loaded patches were then fully dissolved for 15 min in 60 °C water, and total miRNAs were extracted using the miRNeasy serum/plasma advanced Qiagen kits. The expression levels of two endogenous miRNAs (miR-205 and miR-21) were systematically measured by RT-qPCR (Figure 3A). With the exception of HMNs made from the GA-cross-linked PVA/CMC blend, detectable levels of both miRNAs were recovered from all other patches made from either physically or chemically cross-linked materials, with miR-21 systematically found more abundant than miR-205. Variations in absolute miRNA concentrations between patches can be explained by lower degrees of recovery when dissolving HMNs, with some materials breaking down more efficiently than others, with insoluble fibers sometimes found to interfere with the process of miRNA extraction through spin columns.

**Figure 3:**
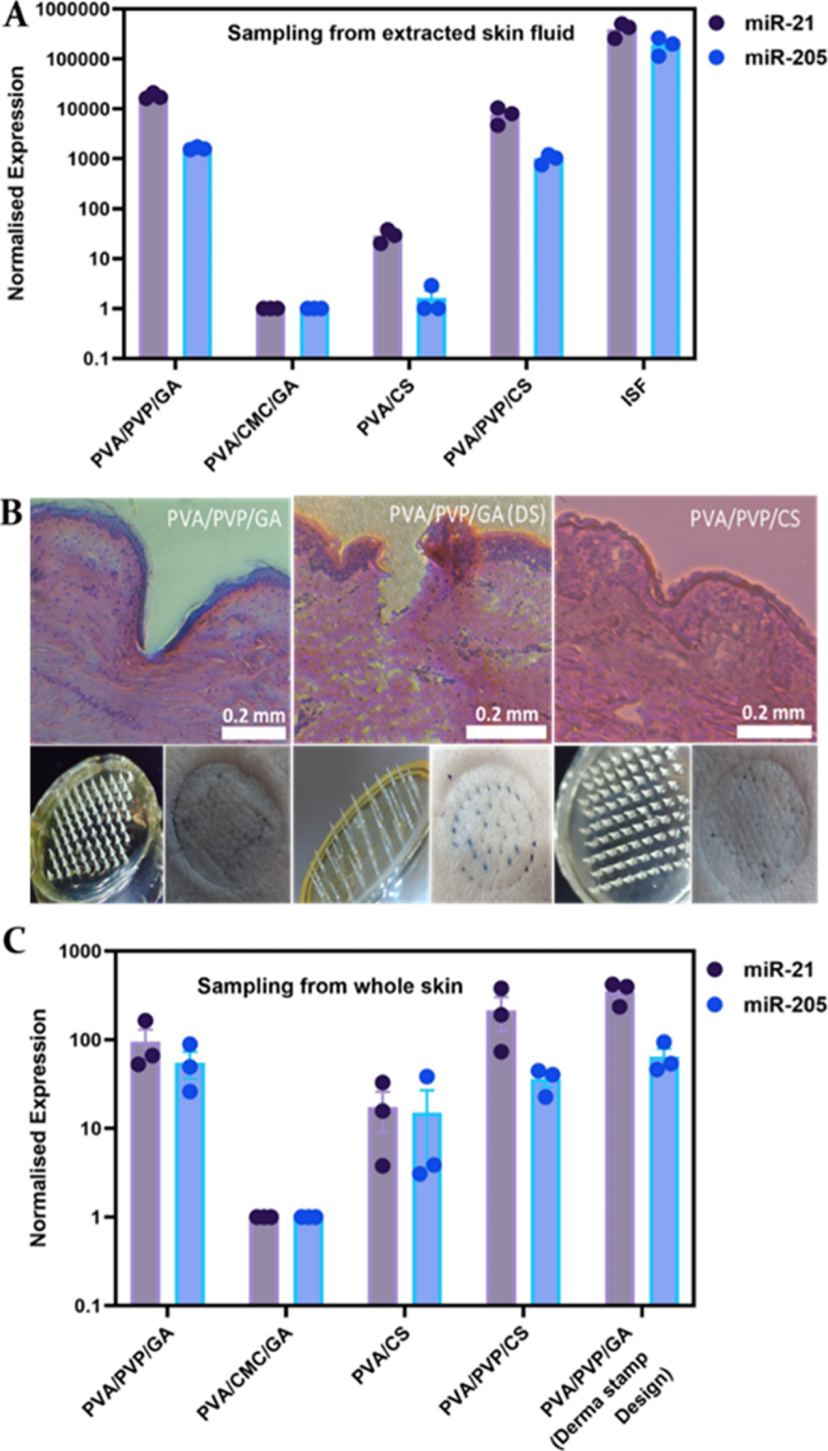
(A) RT-qPCR analysis of two miRNAs (miR-21 and miR-205) from collagenase-extracted ISF from human skin. Four types of HMNs were then used to extract miRNAs from a solution of collagenase extracted ISF, miRNAs recovered from the patches and analyzed by RT-qPCR. Data plotted are normalized 40-CT ± standard error of the mean (*n* = 3). (B) H&E staining of cross sections of HMNs in human skin made with the microneedle frozen in place, and image of the microneedle before insertion alongside an image of human skin after HMNs’ insertion, removal, trypan blue staining, and washing (scale bar 0.2 mm). The first panel shows a PVA/PVP/GA blend made with a 9 × 9 array mold, the second panel shows a PVA/PVP/GA blend in a Derma Stamp (DS) mold, and the third panel shows a PVA/PVP/CS blend made with a 9 × 9 array mold. (C) RT-qPCR analysis of two miRNAs (miR-21 and miR-205) extracted from intact human skin using five types of HMNs (4 different polymer blends, 2 different patch designs). Data plotted are normalized 40-CT ± standard error of the mean (*n* = 3).

#### 3.4.2 Skin Penetration

Epidermal–dermal junction (EDJ) penetration ex vivo was then assessed for three different HMN patches using both H&E staining and trypan blue staining methods (Figure 3B). HMN patches made from either physically cross-linked PVA/PVP/CS blend or GA-cross-linked PVA/PVP blend, using our original design (9 × 9 array of 550 mm needles), were placed onto ex vivo human skin samples and simple thumb pressure applied. Both showed deformation and local stretching of the epidermis but no penetration, as indicated by a lack of trypan blue retention to dermal collagen (Figure 3B, left and right panel). We then compared HMNs made of the same biomaterial but cast in molds with different designs. A PDMS mold was first prepared from a commercially sourced DS (array of 40 0.5 mm needles) and used to prepare HMNs from GA-cross-linked PVA/PVP. When compared to the previous patch design (made from the same material), this time EDJ penetration was clearly observed with both staining methods (Figure 3B, middle panel). This demonstrates the versatility of this biomaterial for the manufacturing of HMN patches of various designs via micro molding.

#### 3.4.3 MiRNA Sampling/Recovery Ex Vivo

The same patches were then pressed onto ex vivo human skin for 5 min, and miRNAs recovered from the patches were analyzed as described in the methods section. PCR-detectable levels of endogenous miR-205 and miR-21 were successfully recovered from all but one patch, with miR-21 concentration consistently higher than that of miR-205, as found in ISF (Figure 3C). The lower concentrations of miRNAs obtained upon applying HMNs on skin can be explained by lower volumes of ISF absorbed this way (21.2 ± 1.2 μL for PVA/PVP/CS system) when compared to ca. 200 μL absorbed by the patch after soaking into extracted ISF. No significant differences in miRNA sampling and recovery were observed when comparing the two HMNs made of the same biomaterial but with different patch designs. This is despite one showing clear rupture of the epidermis (based on the 0.5 mm DS) and the other one (9 × 9 array of 0.55 μm needles) only showing local EDJ stretching. These data suggest that ISF sampling does not strictly require penetration through the EDJ but that other mechanisms such as osmotic pressure can play an important contribution in enabling ISF to diffuse across the skin barrier to the hydrogel matrix.

### 3.5 Biomaterial Biocompatibility

In order to assess the potential cytotoxicity of the biomaterials used for the production of our HMNs, a cell viability assay was used whereby primary human fibroblasts cultured, in vitro, were incubated with HMNs for 10 min (Figure S2). Despite the low amount of GA used during the fabrication process, all HMN patches made from GA-cross-linked polymer blends showed high degree of cellular toxicity, with fibroblast viability dropping below 10%. This is in contrast with the results obtained with physically cross-linked polymer blends (PVA/CS and PVA/PVP/CS) that showed 100% cell viability remaining after incubation with HMNs. Although strategies have been reported to successfully mitigate the toxicity of GA-cross-linked materials,^34–36^ results of all above-mentioned studies combined suggest that the physically cross-linked PVA/PVP/CS blend represents the best material in terms of swelling capacity, mechanical strength, miRNA recovery, and biocompatibility. Only this specific type of HMNs was therefore specifically selected for further in vivo investigations.

### 3.6 Application to Diagnosis of Skin Cancer

With ISF having recently emerged as a valuable source of clinically useful diagnostic and prognostic molecular biomarkers and as an attractive alternative to blood, we used a mouse model of skin cancer to explore the potential application of our HMN platform devised above in healthcare diagnostics in vivo. After 8 weeks, four untreated healthy mice and four carcinogen-treated mice were culled. HMN patches (PVA/PVP/CS blend) were applied on the skin located on the flanks of the mice, in close proximity (within 5 mm) of emerging cSCC skin lesions (premalignancy) and in a similar area on the flanks of age-matched healthy mice. Total miRNAs were extracted as described above and four miRNAs (miR-146b, miR-21, miR-26b, and miR-30d) were selected for targeted RT-qPCR analysis based on the combined results of a hypothesis-free RNA-Seq experiment (Figure 1D) and of a comprehensive literature review on miRNA biomarkers for skin cancers.^6^ miR-21 and miR-146b were highly abundant and overexpressed in our bioinformatic analysis, and this pattern was consistent with HMN sampling. In contrast, miR-30d, which was significantly downregulated bioinformatically, did not show this decrease in our HMN assessment of perilesional ISF. Although miR-26b was abundant in our bioinformatic data set, it was not significantly deregulated at the early time point, despite reports suggesting consistent downregulation within the tumor tissue in SCC, this was not observed in the tissue or serum from our model.^37,38^ Overall, these findings show strong concordance for miR-21 and miR-146b as biomarkers of cSCC measurable by minimally invasive ISF, while additional validation of other candidates is important for translating this technology clinically. For direct comparison, the levels of expression of these four miRNAs were also determined in both serum and whole skin tissue collected from the same mice receiving the HMN patches (Figure 4).

**Figure 4:**
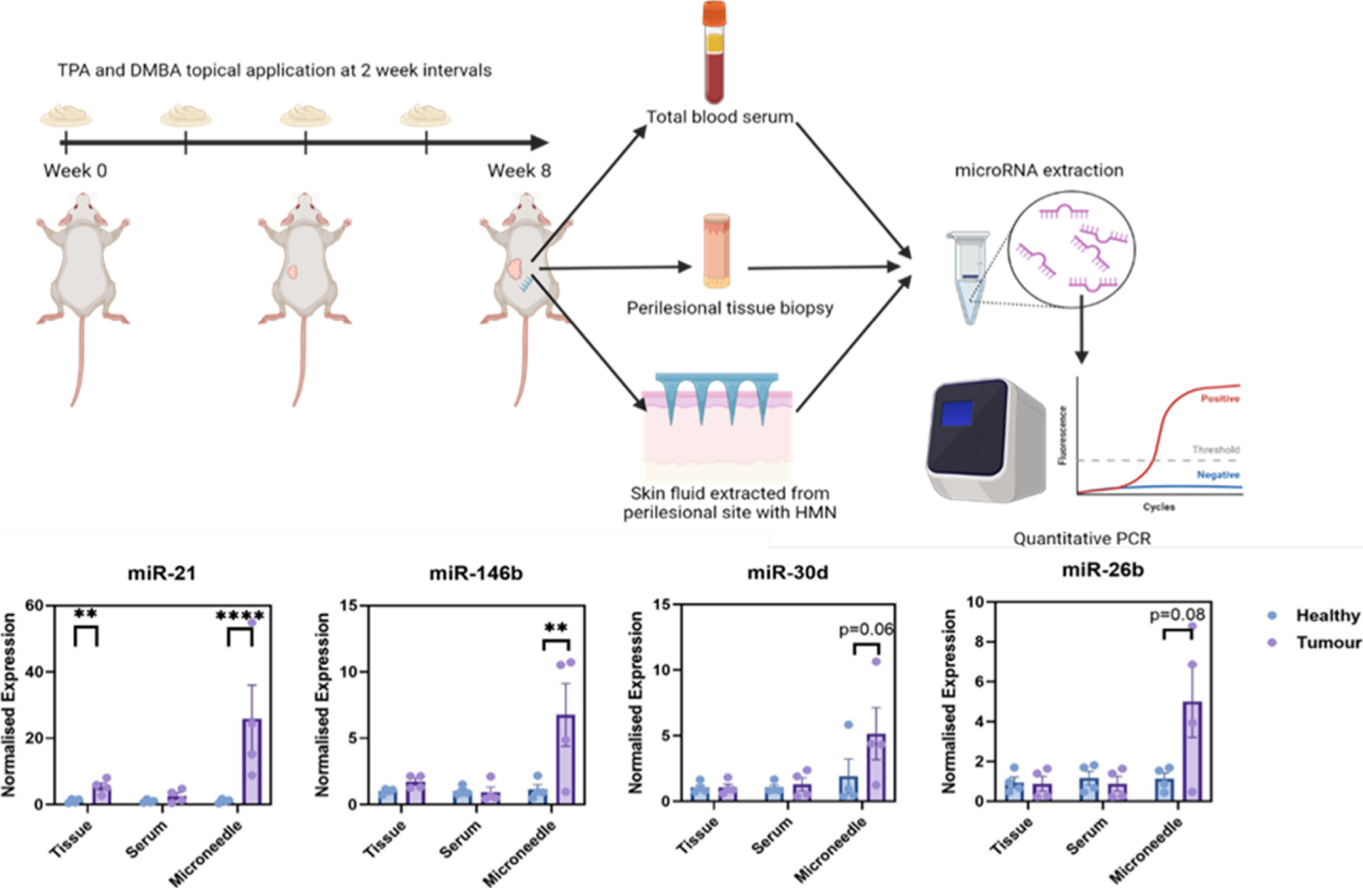
The schematic represents the experimental workflow for inducing and sampling from mice. Mice were treated with DMBA/TPA to induce cSCC (*N* = 4/grp) or left untreated. After 8 weeks mice were culled by exsanguination, total bloods collected and spun down for serum collection and subsequent RNA extraction. The back skin was excised, kept on ice until HMN was applied for 5 min, and a 1 mm punch biopsy was taken for total tissue RNA. The figure below shows RT-qPCR analysis of four miRNAs extracted from tissue, blood serum, or HMNs within 5 mm of the tumor site. U6 expression was used to calculate ΔCT and data plotted are relative to naive mice. A two-way ANOVA, with Sidak’s multiple comparison, was performed on ΔCT values for each miRNA, on Graphpad Prism 10. *P* value ** <0.01, ****0.0001.

While miR-21 was found to be significantly up-regulated in tissues of carcinogen-treated mice compared to naïve mice (suggesting some inflammation of the skin resulting from the direct exposure to carcinogenic chemicals), a much greater level of upregulation was observed in skin fluid collected with HMNs. No statistically significant differences were observed between tissues from cancer and healthy mice for the other three miRNAs, which can be explained by the fact that, even in the case of cSCC mice, tissues were collected only in proximity of emerging skin lesions. Above 4-fold upregulation of all four miRNAs was observed in skin fluid of cancer mice compared to healthy controls, with strong or near statistical significance. This is in striking contrast with the absence of any statistically significant differences when interrogating serum. These results strongly support our initial hypothesis that slowly diffusing extracellular fluids sampled perilesionally contain greater levels of tumor-derived or tumor-associated molecular biomarkers than bodily fluids in rapid systemic circulation. These results paired with the marked increase in perilesional miR-21 and miR-205 (Figure 1A) demonstrate that in a translational setting HMNs would be applied next to the suspected lesion for sampling and subsequent miRNA quantification and a healthy site could also be sampled for individual baseline measures for each patient. These results also validate our bespoke HMNs as minimally invasive and biocompatible platforms for interrogating skin fluids with a unique spatiotemporal resolution.

## 4 Conclusion

In summary, we have used micro molding to develop a new generation of HMN patches made of a noncytotoxic, mechanically strong, highly absorbent hydrogel from a physically cross-linked blend of three copolymers, PVA/PVP/CS. Using human skin samples, we have demonstrated that ISF, and ubiquitous analytes within it, could be sampled within minutes without strict requirements for penetration through the epidermis. Critically for biomedical applications, miRNAs were successfully recovered after sampling to enable semiquantitative analysis by RT-qPCR. Using an in vivo mouse model of cSCC we also demonstrated that HMNs applied near newly emerging skin lesions could distinguish between mice developing cSCC and healthy skin tissue based on the detection of a panel of miRNAs heavily deregulated in ISF. Besides miR-21, a pan-cancer biomarker already well established in the literature,^39^ we show for the first time that miRNAs including miR-146b, miR-30d, and miR-26b that were previously associated with oral squamous cell carcinoma,^40^ cervical squamous cell carcinoma,^41^ and esophageal squamous cell carcinoma^42^ are specifically deregulated in ISF of cSCC mice. Strikingly, no statistically significant differences in the relative expression of these four miRNAs were found when interrogating the serum, validating ISF as a promising alternative to blood for the early diagnosis of skin cancers. Unlike blood, which is freely circulating throughout the entire body and will only contain highly diluted amounts of tumor-derived or tumor-associated biomarkers, interstitial fluid is characterized by a much slower diffusion, leading to biomarkers sampled locally to be much more representative of the microenvironment. By applying HMNs within close distance of cancerous skin lesions, it is therefore possible to capture tumor-associated miRNAs before they enter the systemic circulation, resulting in a greater sensitivity to detect early signs of skin dysregulation. This is of high significance because it could potentially simplify the detection of abnormally expressed miRNAs, even in the early stages of a disease when the levels of miRNA deregulation are low and challenging to detect in highly diluted environments, such as blood. Medical devices based on this technology could therefore emerge that can improve the stratification of skin cancer patients in primary care settings.

## Supplementary Material



## Notes

Raw RNA-Seq files will be provided by the authors upon request. RNA-Seq data and results of cytotoxicity experiments (PDF)
