# Supplementary material for: MiRNAs in Interstitial Skin Fluid Sampled with Swellable Hydrogel Microneedles Are Locally Deregulated Near Malignant Skin Lesions in Early Stages of Cutaneous Squamous Cell Carcinoma
Source: ACS Biomater Sci Eng. 2026 Jan 22;12(2):1245–55. doi: 10.1021/acsbiomaterials.5c01505 (PMC12892239; doi:10.1021/acsbiomaterials.5c01505)

Supporting Information

MiRNAs in interstitial skin fluid sampled with swellable hydrogel microneedles are locally deregulated near malignant skin lesions in early stages of cutaneous squamous cell carcinoma

Ahmad Kenaan,[1†] Oliver Teenan,[1†] Connor Daniels,[1] Christina Malaktou,[2] Mo Akhavani,[3] Nikolaos Sideris[4], Leandro Castellano[4], Jessica Strid,[2] Claire A. Higgins[1] and Sylvain Ladame*[1]

[1]Department of Bioengineering, Imperial College London, White City Campus, London, W12 0BZ, UK.

[2]Department of Immunology and Inflammation, Imperial College London, Hammersmith Campus, London, W12 0NN, UK.

[3]The Plastic Surgery Group, 100 Harley Street, Marylebone, London W1G 7JA.

[4]Department of Biochemistry, University of Sussex, Brighton, CN1 9QJ UK

Figure S1: Mean of normalised counts vs log fold change for each miRNA identified by small RNA sequencing in ISF. **A)** Comparison of week 12 tumour skin vs naïve ISF. Colour scale indicates p. value, blue-green non-significant, yellow p. value <0.05, calculated by DESeq2. **B)** Comparison of week 17 tumour ISF vs naïve. **C)** Comparison of week 12 vs week 17 tumour ISF. **D)** comparison of all tumour ISF timepoints grouped vs naïve skin. **E)** Box plots of normalised counts for mmu-miR-21a in ISF. **F)** Box plot of normalised counts for mmu-miR-146b in ISF.

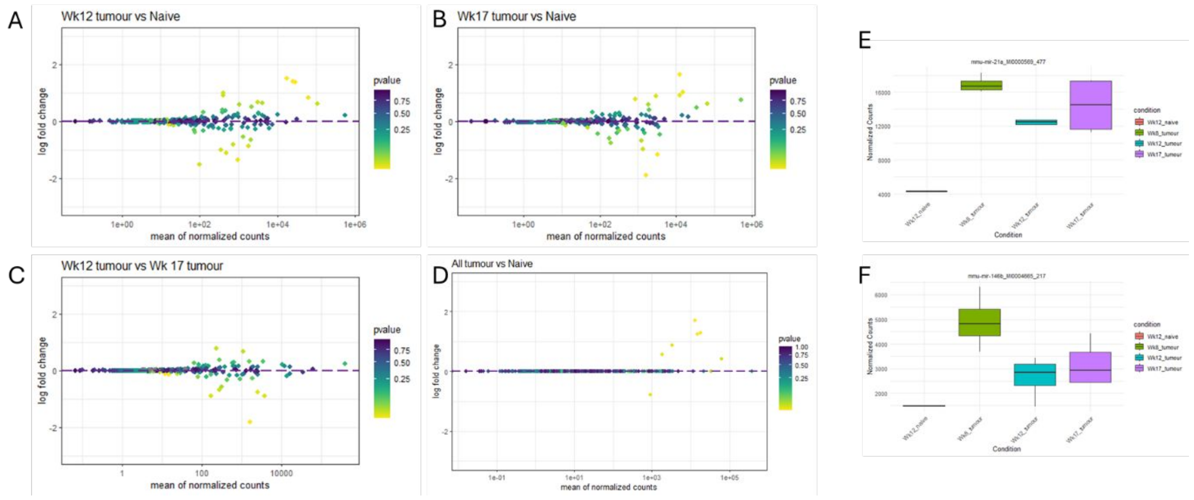

Figure S2: Cell viability measurements indicate that PVA/CS and PVA/PVP/CS patches, utilizing chitosan with physical crosslinking, exhibit no toxicity with 100% cell viability, while PVA/PVP/GA and PVA/CMC/GA patches show high toxicity when GA is used as a chemical crosslinker.

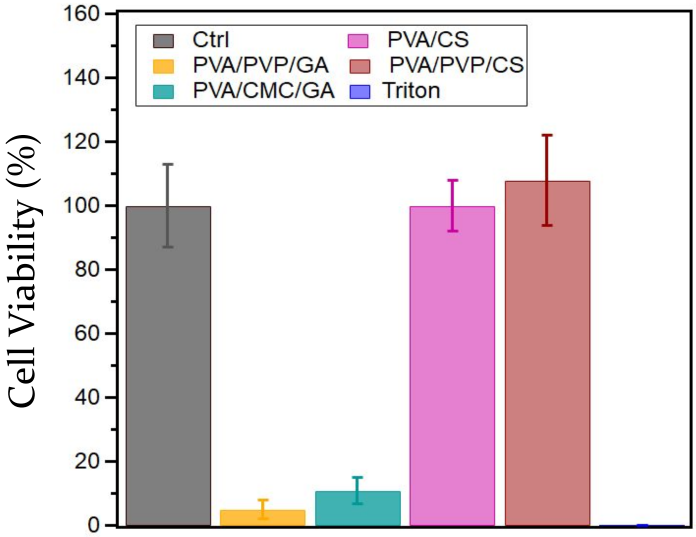

Supplement: Supplementary file 1 [file ab5c01505_si_001.pdf]
